# Response to Commentary

**DOI:** 10.31138/mjr.240126.rco

**Published:** 2026-03-01

**Authors:** Mahabaleshwar Mamadapur, Ramaswamy Subramanian

**Affiliations:** Clinical Immunology and Rheumatology, JSS Medical College, Karnataka, India

Dear Editor,

Thank you for the opportunity to respond to the letter regarding our case series on Methotrexate-induced accelerated nodulosis (MIAN).^1^ We appreciate the reviewers’ critical appraisal and valuable insights.

We carefully reviewed the commentary on the estimated prevalence of MIAN. We acknowledge that the figure of 8–11.6% cited in our discussion was derived from historical literature, specifically Kremer et al.,^2^ Kerstens et al.,^3^ Patatanian et al.,^4^ and Agarwal et al.^5^. The studies cited in this context were intended to illustrate the magnitude of MIAN observed in selected cohorts, rather than to claim a population-level rate applicable to the current RA population.

The historical series cited involves small, selected cohorts with variable disease activity, different background therapies, and some case reports with uncertain causality as assessed using structured scales. These limitations reduce the scope of generalisability. We agree that modern randomised clinical trials have not reported similar high frequencies of MIAN. But, at the same time, MIAN may be underrepresented due to several reasons, like variable case definitions, delayed onset and lack of systemic/dermatologic surveillance as adverse effects. We studied the TREAT EARLIER^6^ and CIRT^7^ trials and concluded that, although the sample sizes were large, MIAN surveillance was not reported in the adverse effects/secondary endpoints. It would be interesting if future trials or original studies considered MIAN as a surveillance marker or endpoint. We agree that methotrexate remains an anchor drug for the management of RA in resource-limited settings. However, MIAN remains a relevant clinical entity requiring vigilance.

To avoid misunderstandings in future versions, we propose the following word replacement.

MIAN is an uncommon but well-recognised complication of methotrexate therapy. Historical case series have reported cases in selected cohorts (approximately 8–12%), but these figures reflect selected populations and variable case definitions. Contemporary RCTs have not systemically captured MIAN and, therefore, cannot conclude its exact prevalence.

We thank the authors of the second letter^8^ for their valuable diagnostic and therapeutic insights. We strongly agree with their observations regarding the dissociation between systemic inflammation and nodulogenesis, as well as the risk factors and features that differentiate MIAN from rheumatoid nodules. We welcome the proposed clinical algorithm for the evaluation and management of MIAN. We support the call for multi-centre, prospective studies that will help fill research gaps.
